# The Physician’s Visiting List

**Published:** 1894-01

**Authors:** 


### The Physician's Visiting List. (Lindsay & Blackiston's).

The fact that this Visiting List has been published annually for forty years is sufficient guarantee of its excellence and popularity. In addition to the visiting list proper, it contains easily-accessible suggestions upon many of the emergencies that may arise in a physician's practice, as when he is too far from home to learn from his text-books the antidote for a poison that may have been swallowed or the proper method of resuscitating a half-drowned person. True, he should know these things, but who does not occasionally forget, when he most wishes to remember? There are also dose-tables, tables of the metric system, a list of new remedies, rules for examining urine, a table for calculating the period of pregnancy, and other equally useful information. The arrangement for entering patients, visits, consultations, etc., is exceedingly simple, and the whole makes a thin, compact, and easily-carried volume.

PRICES OF REGULAR EDITION.

| | | | | | | | |
|---|---|---|---|---|---|---|---|
| For 25 | Patients | per day | or week, | | Pencil, pockets, etc., | | $1.00 |
| 50 | " | " | " | | " " " | | 1.25 |
| 75 | " | " | " | | " " " | | 1.50 |
| 100 | " | " | " | | " " " | | 2.00 |
| 50 | " | " | " | 2 Vols. | Jan. to June<br>July to Dec. | | 2.50 |
| 100 | " | " | " | 2 Vols | Jan. to June<br>July to Dec. | | 3.00 |

Address P. Blakiston, Son & Co., 1012 Walnut Street, Philadelphia.

